# Confirmation and Identification of Biomarkers Implicating Environmental Triggers in the Pathogenesis of Type 1 Diabetes

**DOI:** 10.3389/fimmu.2020.01922

**Published:** 2020-09-15

**Authors:** Robert Z. Harms, Katie R. Ostlund, Monina S. Cabrera, Earline Edwards, Marisa Fisher, Nora Sarvetnick

**Affiliations:** ^1^Department of Surgery-Transplant, University of Nebraska Medical Center, Omaha, NE, United States; ^2^Endocrine Clinic, Children's Hospital and Medical Center, Omaha, NE, United States; ^3^Mary and Dick Holland Regenerative Medicine Program, University of Nebraska Medical Center, Omaha, NE, United States

**Keywords:** Type 1 diabetes (T1D), acute phase proteins (APP), Vitamin D, virus, cytokine, EndoCAbs, adipokines, human blood plasma

## Abstract

Multiple environmental triggers have been proposed to explain the increased incidence of type 1 diabetes (T1D). These include viral infections, microbiome disturbances, metabolic disorders, and vitamin D deficiency. Here, we used ELISA to examine blood plasma from juvenile T1D subjects and age-matched controls for the abundance of several circulating factors relevant to these hypotheses. We screened plasma for sCD14, mannose binding lectin (MBL), lipopolysaccharide binding protein (LBP), c-reactive protein (CRP), fatty acid binding protein 2 (FABP2), human growth hormone, leptin, total adiponectin, high molecular weight (HMW) adiponectin, total IgG, total IgA, total IgM, endotoxin core antibodies (EndoCAbs), 25(OH) vitamin D, vitamin D binding protein, IL-7, IL-10, IFN-γ, TNF-α, IL-17A, IL-18, and IL-18BPa. Subjects also were tested for prevalence of antibodies targeting adenovirus, parainfluenza 1/2/3, Coxsackievirus, cytomegalovirus, Epstein-Barr virus viral capsid antigen (EBV VCA), herpes simplex virus 1, and *Saccharomyces cerevisiae*. Finally, all subjects were screened for presence and abundance of autoantibodies targeting islet cell cytoplasmic proteins (ICA), glutamate decarboxylase 2 (GAD65), zinc transporter 8 (ZNT8), insulinoma antigen 2 (IA-2), tissue transglutaminase, and thyroid peroxidase, while β cell function was gauged by measuring c-peptide levels. We observed few differences between control and T1D subjects. Of these, we found elevated sCD14, IL-18BPa, and FABP2, and reduced total IgM. Female T1D subjects were notably elevated in CRP levels compared to control, while males were similar. T1D subjects also had significantly lower prevalence of EBV VCA antibodies compared to control. Lastly, we observed that c-peptide levels were significantly correlated with leptin levels among controls, but this relationship was not significant among T1D subjects. Alternatively, adiponectin levels were significantly correlated with c-peptide levels among T1D subjects, while controls showed no relationship between these two factors. Among T1D subjects, the highest c-peptide levels were associated with the lowest adiponectin levels, an indication of insulin resistance. In total, from our examination we found limited data that strongly support any of the hypotheses investigated. Rather, we observed an indication of unexplained monocyte/macrophage activation in T1D subjects judging from elevated levels of sCD14 and IL-18BPa. These observations were partnered with unique associations between adipokines and c-peptide levels among T1D subjects.

## Introduction

The clinical definition of type 1 diabetes is not overly controversial. The absence of insulin production and presence of circulating antibodies targeting islet cell-associated proteins (i.e., islet autoantibodies) are sufficient to outline the disease. However, the mechanism(s) whereby beta cells are ultimately destroyed remains unclear and highly contested. Leaving aside known genetic risk associations (1), there remains an abundant number of hypotheses as to what triggers drive pathogenesis. The range of suspected triggers is diverse, including viral infection (2), microbiome disturbances and related gut “leakiness” (3, 4), metabolic disorder (5), vitamin D deficiency (6), and dysregulated immunity (7, 8). Adding to this broad range is the high likelihood that these factors can easily interact with one another, playing upon known genetic predisposal to produce an exceedingly complex etiology.

In the face of such an extensive array of probable causality, one enticing option is to investigate each hypothesis. This approach would avoid a bias of favoritism while the issue remains contested, and would allow for the observation of multifactorial causation, if apparent. In pursuing this approach, one is first confronted with two related concerns: method and scope. While pancreas and intestinal biopsies would be most appropriate for these studies, access to these tissues is highly limited, and their acquisition outside of the postmortem condition can come with some risk (9). Alternatively, peripheral blood offers a low risk and more readily available tissue that can be separated into cellular and plasma components for study. Containing a rich assemblage of soluble factors, peripheral blood plasma provides an intricate mosaic of internal health, capable of revealing the turbulent and heterogeneous immunological landscape to the investigator. To assess this terrain, multiplex assays and mass spectrometry have been used in the investigation of T1D previously (10, 11). While these methods can be suitable depending on the question, they are not appropriate or available for all targets (e.g., antibodies targeting viral or islet proteins) and require specialized instrumentation for analysis. For another option, enzyme linked immunosorbent assay (ELISA) is a well-established and reliable alternative in analyte analysis. Although more labor intensive since only one analyte can be measured at once, it is methodologically robust. Furthermore, since plate readers are commonly available, results can be independently confirmed at laboratories worldwide without unreasonable investments.

With respect to the concern of scope, a vast number of potential targets in the plasma could be considered relevant to address these questions. Here, with few exceptions, we directed our analysis to selected targets that had been investigated previously and deemed relevant in T1D and/or autoimmunity. These are described more fully below. By querying several causal hypotheses, our goal was to work toward a more thorough evaluation of immunity among diagnosed T1D subjects to establish potential inroads in therapy and solidify purported avenues of pathogenesis. Ultimately, the majority of the factors we measured were similarly abundant in T1D subjects and controls, yet we did observe a handful of novel differences. These included increased abundance of sCD14 and IL-18BPa and unique associations between adipokines and c-peptide among T1D subjects. Albeit in instances provocative, and certainly with limitations, our findings did not strongly support any of the tested hypotheses; rather, they provided hints and suggestions for future queries.

## Methods

### Study Background and Consent

We are performing a collaborative study with researchers and clinicians at Children's Hospital and the University of Nebraska Medical Center designed to explore how genetic and environmental variables influence the development of type 1 diabetes. We actively recruited diagnosed type 1 diabetic juveniles and control (no history or family history of autoimmunity, short stature diagnosis) juveniles from the Endocrine Clinic at Children's Hospital. The study was performed under ethical standards defined by the UNMC Institutional Review Board (IRB# 107-09-EP) and in accordance with the 1964 Declaration of Helsinki. All subjects who participated provided informed consent/assent. Subject characteristics are provided in Table 1.

**Table 1:** Patient data.

	**Control**	**T1D**	***p*-value**
*n* (*n* female)	27 (18)	36 (18)	
Age			
Mean	9.81	9.47	0.248
Range	8–11	8–11	
HbA1c			
Mean	n/a	8.81	n/a
Range	n/a	6.1 - 15.1	
BMI			
Mean	17.5	20.1	0.012
Range	13.4–30.4	13.7–31.6	
Months since diagnosis			
Mean	n/a	21.8	n/a
Range	n/a	0–88	
Race/Ethnicity			
American Indian	0	1	
Asian	2	0	
Black American	2	1	
Hispanic	4	3	
White	18	30	
Other	1	1	

### Sample Processing

We acquired 20 mL of venous blood in K_2_ EDTA vacutainers (Becton, Dickinson, and Company). Blood samples were processed within 2 h of blood draw. For plasma isolation, whole blood tubes were centrifuged at 400 × g for 20 min at 18°C. Platelet-rich plasma was removed and centrifuged for 10,000 × g for 10 min at 4°C. Supernatents were then pooled and individual aliquots were flash frozen with dry ice, then stored at −80°C. To avoid multiple freeze-thaw cycles, aliquots were thawed and subdivided into assay-specific volumes, and then refrozen as described above.

### ELISA

The following ELISAs were used according to manufacturers' recommendations: from R & D systems sCD14 (DC140), MBL (DMBL00), CRP (DCRP00), Vitamin D BP (DVDBP0), FABP2 (DFBP20), total adiponectin (DRP300), HMW adiponectin (DHWAD0), leptin (DLP00), high sensitivity IL-7 (HS750), and IL-18BPa (DBP180); from Enzo BioChem human growth hormone (HGH, KIT148-0001) and 25(OH)Vitamin D (ADI-900-215); from Hycult Biotech lipopolysaccharide binding protein (LBP, HK315) and EndoCAb IgM, IgA, and IgG (HK504-IGG, -IGA, -IGM); from eBiosience/ThermoFisher total IgA (88–50600), total IgG IgG(88–50550), total IgM (88–50620), IL-18 (BMS267), high sensitivity IL-17A (BMS2017HS), high sensitivity TNF-α (BMS223HS), high sensitivity IL-10(BMS215HS), and high sensitivity IFN-γ (BMS228HS); from AbCam MDC (ab223866); from Eagle Biosciences GAD65(GAD31-K01), ICA (ICA31-K01), ZnT8 (ZT831-K01), IA-2 (IA231-K01), tTG IgA (HTG31-K01), and TPO (TPO31-K01); from Generic Assays ASCA (4006); from IBL America parainfluenza 1/2/3 IgG (IB79269), adenovirus IgG (IB79202), EBV VCA IgG (RE57351), CMV IgG (EG 101), HSV1 IgG (IB79242), Coxsackievirus IgG (IB05040), echovirus IgG (IB05049); from Alpco C-peptide (80-CPTHU-E01.1) for random, non-fasting measurement (12). Absorbance values were measured using a Biotek H2 Hybrid plate reader (BioTek). Standard curves were modeled using Gen5 software v 3.05.11 (BioTek). Sample values below the limit of detection (LOD) were given a synthetic value equaling the LOD/$\sqrt{2}$. For IFN-γ, 1 control and 3 T1Ds were < LOD; for TNF-α, all subjects tested were < LOD; for IL-17A, 26 controls and 32 T1Ds were < LOD.

### Statistics

For pairwise comparisons, sample values were normalized using natural log transformations and then tested for significant differences using the two-tailed Student's *t*-test. Correlations were explored using Pearson's product moment correlation test on normalized values. Months since diagnosis values, which contained true zeroes, were normalized using square root transformations for these tests. For comparisons of pathogen prevalence, Fisher's exact test was used. In all cases, a result was considered significant if *p* < 0.05. When present, exploratory sex stratification is intended to help address the issue of how sex influences health and disease (13, 14). Statistical tests were performed using SPSS v 25 (IBM) and Prism v 6.03 (Graphpad). Figures were constructed using Prism. Non-transformed values were depicted for pairwise comparisons to facilitate interpretation. Descriptive statistics for analyte values are provided in Supplementary Data 1.

## Results

### Elevated Autoantibodies and Decreased C-Peptide Among T1D Subjects

**Figure 1:**
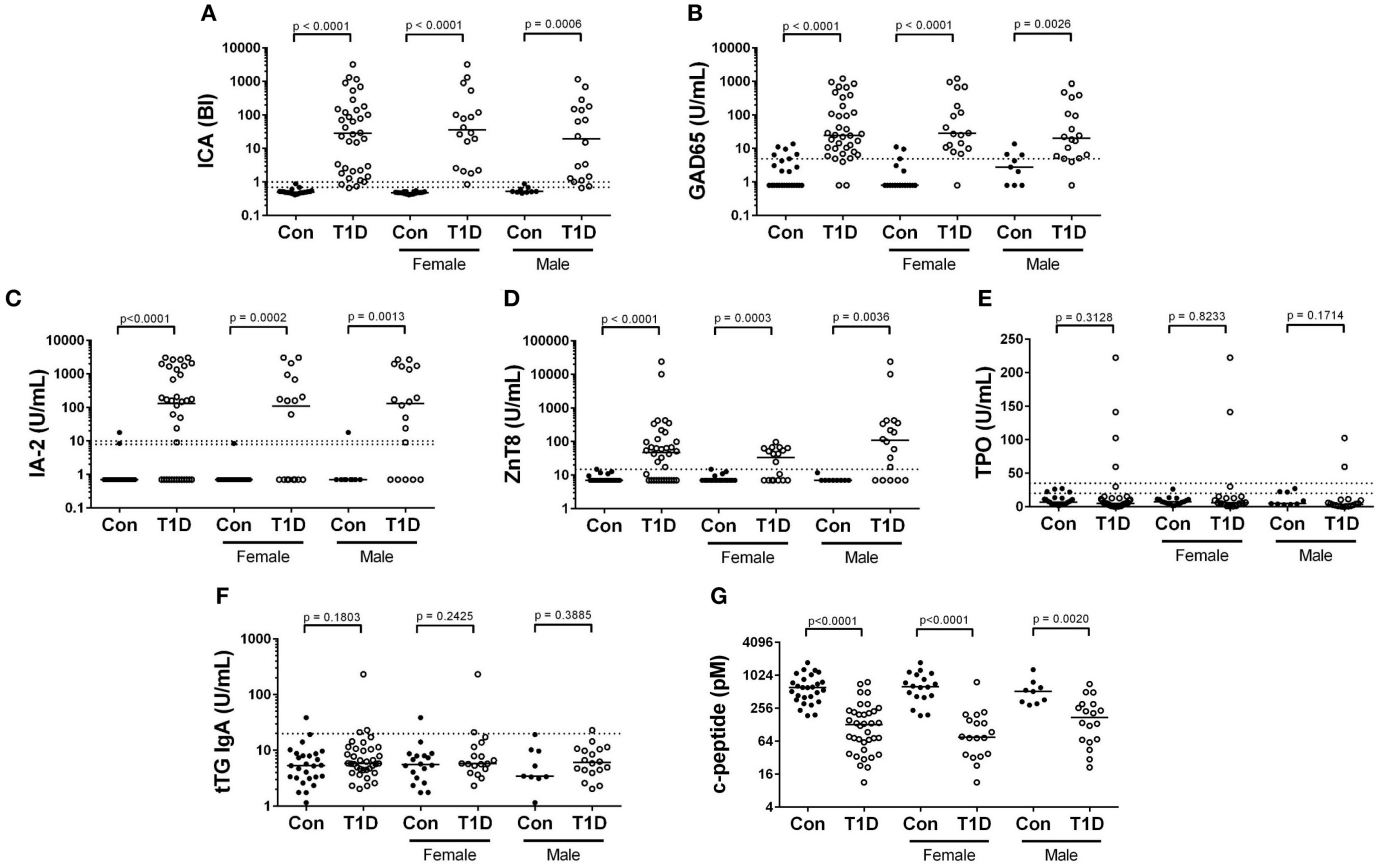
Autoantibody and c-peptide levels among diagnosed T1D subjects and controls. **(A–D)** Abundance of islet cell antibodies (ICA), glutamate decarboxylase 2 (GAD65), insulinoma antigen 2 (IA-2), and zinc transporter 8 (ZnT8), were all significantly elevated among T1D subjects, consistent with the disease classification. **(E,F)** We observed no significant difference in abundance of antibodies targeting thyroperoxidase (TPO) and tissue transglutaminase (tTG), both of which have been previously associated with type 1 diabetes. **(G)** Random, non-fasting c-peptide levels were significantly decreased among T1D subjects compared to controls. Bars represent median values and BI signifies binding index. Dotted lines on autoantibody graphs represent cutoff points for designating positive or negative status.

The presence of circulating autoantibodies targeting islet-associated antigens and an absence of insulin are hallmarks of T1D. We compared our control and T1D subjects for presence and abundance of islet cell antibodies (ICA), glutamate decarboxylase 2 (GAD65), zinc transporter 8 (ZnT8), and insulinoma antigen 2 (IA-2, or receptor-type tyrosine-protein phosphatase-like N). The results are presented in Figure 1 and qualitatively summarized in Table 2. As expected, clinically-diagnosed T1D subjects were significantly increased for all islet-associated autoantibodies compared to control (Figures 1A–D). Interestingly, autoantibodies were also detected among controls with 5 of 27 having GAD65, 1 of 27 with ZnT8, and 1 of 27 with IA-2. No control subjects were positive for ICA.

**Table 2:** Qualitative autoantibody assessment.

	**Positive/Gray/Negative (% positive)**		
	**Combined**	**Female**	**Male**
ICA			
Control	0/1/26 (0)	0/0/18 (0)	0/1/8 (0)
T1D	33/2/1 (92)	17/1/0 (94.4)	16/1/1 (88.9)
GAD65			
Control	5/0/22 (22.3)	2/0/16 (11.1)	3/0/6 (33.3)
T1D	32/0/4 (88.9)	17/0/1 (94.4)	15/0/3 (83.3)
IA-2			
Control	1/1/25 (3.7)	0/1/17 (0)	1/0/8 (11.1)
T1D	22/1/13 (61.1)	10/0/8 (55.6)	12/1/5 (66.7)
ZnT8			
Control	1/0/26 (3.7)	1/0/17 (5.6)	0/0/9 (0)
T1D	23/0/13 (63.9)	10/0/8 (55.6)	13/0/5 (72.2)
TPO			
Control	0/4/23 (0)	0/1/17 (0)	0/3/6 (0)
T1D	4/1/31 (11.1)	2/1/15 (11.1)	2/0/16 (11.1)
tTG IgA			
Control	1/0/26 (3.7)	1/0/17 (5.6)	0/0/9 (0)
T1D	3/0/33 (8.3)	2/0/16 (11.1)	1/0/17 (5.6)

Anti-thyroid peroxidase (TPO)antibodies have been reported to be elevated in T1D and could indicate or prognose thyroid autoimmunity (15, 16). We detected TPO autoantibodies in only 4 of 36 T1D subjects and in no controls (Figure 1E, Table 2). Tissue transglutaminase (tTG) IgA antibodies are associated with celiac disease, and are not uncommonly detected in juvenile T1D subjects (17). We detected tTG IgA autoantibodies in 1 control and 3 T1D subjects (Figure 1F, Table 2). Importantly, tTG levels are negatively associated with gluten-free diets. As we do not know our subjects dietary regimen, we may be underestimating the ability of our subjects to produce these antibodies. Finally, we observed significantly and drastically reduced random, non-fasting c-peptide levels among T1D subjects compared to controls (Figure 1G). Intriguingly, a handful of T1D subjects had c-peptide levels which were similar to control subjects. These are explored further below.

### Viral Immunoglobulins

Exposure to viruses and other pathogens frequently elicits a humoral response which is detectable by measuring antigen-specific immunoglobulins in plasma. This lasting response is a fundamental component of successful adaptive immunity. This measurement can provide a murky “history” of previous viral infection, and virus exposure has been hypothesized as a causal factor in T1D development. We examined T1D and control plasma for levels of IgG specific for adenovirus, parainfluenza 1/2/3, Epstein-Barr virus viral capsid antigen (EBV VCA), cytomegalovirus (CMV), Coxsackievirus, echovirus, herpes simplex virus 1 (HSV1), and IgA to *Saccharomyces cerevisiae*. The qualitative results from these analyses are presented in Table 3. Antibodies targeting the common respiratory viruses adenovirus and parainfluenza 1/2/3 were detected in nearly all T1D and control subjects. Prevalence of Coxsackievirus IgG positive individuals was relatively similar between controls and T1D subjects. Coxsackievirus IgG was detected in 9 of 27 (33.3%) controls and 10 of 36 (27.8%) T1D subjects. Highly similar results were obtained for IgG targeting echovirus (Supplemental Data 2), another member of the genus *Enterovirus*. Ignoring disease classification, female prevalence of Coxsackievirus IgG was significantly higher (*p* < 0.0001) with 18/36 (50%) females possessing detectable Coxsackiesvirus IgG, and only 1/27 (3.70%) in males. Among the herpes viruses, EBV VCA IgG prevalence was significantly lower among T1D subjects compared to controls (*p* = 0.0409), with EBV VCA IgG detected in 20 of 27 (74.1%) of controls and 17 of 36 (47.2%) with T1D. CMV IgG prevalence was also lower in T1D, yet this was not significant (*p* = 0.2507). CMV IgG was detected in 9 of 27 (33.3) controls and 7 of 36 T1D subjects (19.4%). HSV1 IgG was detected in 3 of 27 (11.1%) controls and 9 of 36 (25%) with T1D. Although the prevalence was higher in T1D, this did not reach significance (*p* = 0.2069), but approached significance in males (*p* = 0.0593). Finally, we were unable to detect *S. cerevisiae* IgA (ASCA), a marker associated with some forms of inflammatory bowel disease (18), in any subjects.

**Table 3:** Qualitative anti-pathogen antibody assessment.

	**Positive/Gray/Negative (% positive)**		
	**Combined**	**Female**	**Male**
Adenovirus IgG			
Control	27/0/0 (100)	18/0/0 (100)	9/0/0 (100)
T1D	36/0/0 (100)	18/0/0 (100)	18/0/0 (100)
ASCA IgA			
Control	0/0/26 (0)	0/0/17 (0)	0/0/9 (0)
T1D	0/0/36 (0)	0/0/18 (0)	0/0/18 (0)
Coxsackievirus IgG			
Control	9/2/16 (33.3)	9/1/8 (50)^†^	*0/1/8 (0)^†^*
T1D	10/4/22 (27.8)	9/2/7 (50)^†^	*1/2/15 (5.9)^†^*
CMV IgG			
Control	9/0/18 (33.3)	7/0/11 (38.9)	2/0/7 (22.2)
T1D	7/0/29 (19.4)	4/0/14 (22.2)	3/0/15 (16.7)
EBV VCA IgG			
Control	20/0/7 (74.1)^*^	14/0/4 (77.8)	6/0/3 (66.7)
T1D	17/0/19 (47.2)^*^	9/0/9 (50)	8/0/10 (44.4)
HSV 1 IgG			
Control	3/0/24 (11.1)	3/0/15 (16.7)	0/0/9 (0)^#^
T1D	9/0/27 (25)	2/0/16 (11.1)	7/0/11 (38.9)^#^
Parainfluenza 1/2/3 IgG			
Control	24/2/1 (88.9)	17/0/1 (94.4)	7/2/0 (77.8)
T1D	31/4/1 (86.1)	16/2/0 (88.9)	15/2/1 (83.3)

† *p < 0.0001 when pooling total females (T1D and control) and comparing to total males (T1D and control)*.

* *p = 0.0409 comparing combined T1D and combined control*.

# *p = 0.0593 comparing male T1D and male control*.

*ASCA, anti-Saccharomyces cerevisiae antibodies*.

### Decrease in Abundance of EndoCAb IgG With Time Since Diagnosis

**Figure 2:**
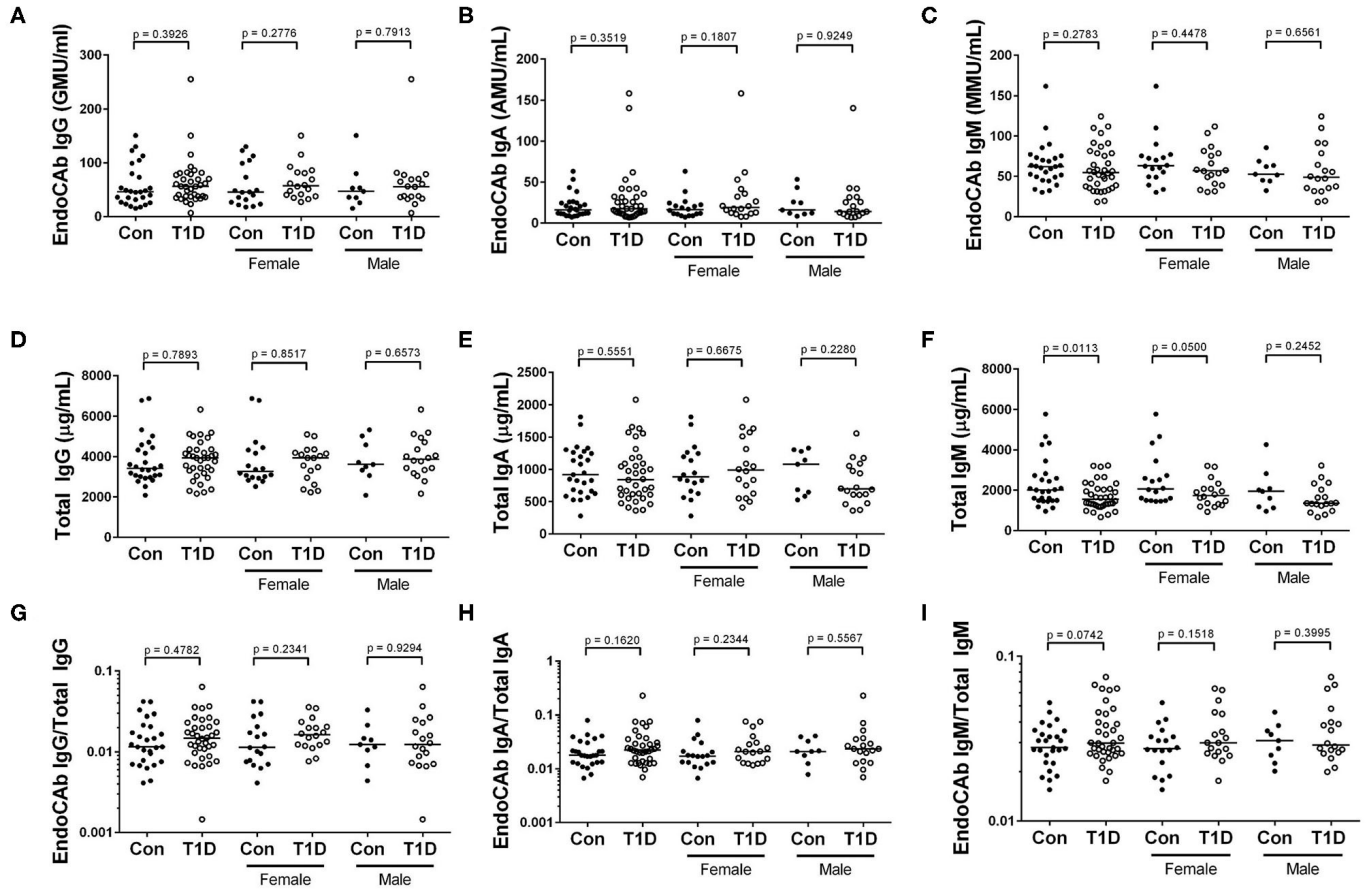
Reduced IgM abundance among T1D subjects, while endotoxin core antibodies (EndoCAb) and total IgG and IgA levels are similar to control. **(A–C)** EndoCAb IgG, IgA, and IgM levels were similar among T1D subjects and controls. **(D–F)** We found that total IgG and total IgA were also similar, while total IgM was reduced among T1D subjects. **(G–I)** Finally, the ratio of EndoCAbs to total immunoglobulins were more-or-less equivalent for IgG, IgA, and IgM. Bars represent median values.

**Figure 3:**
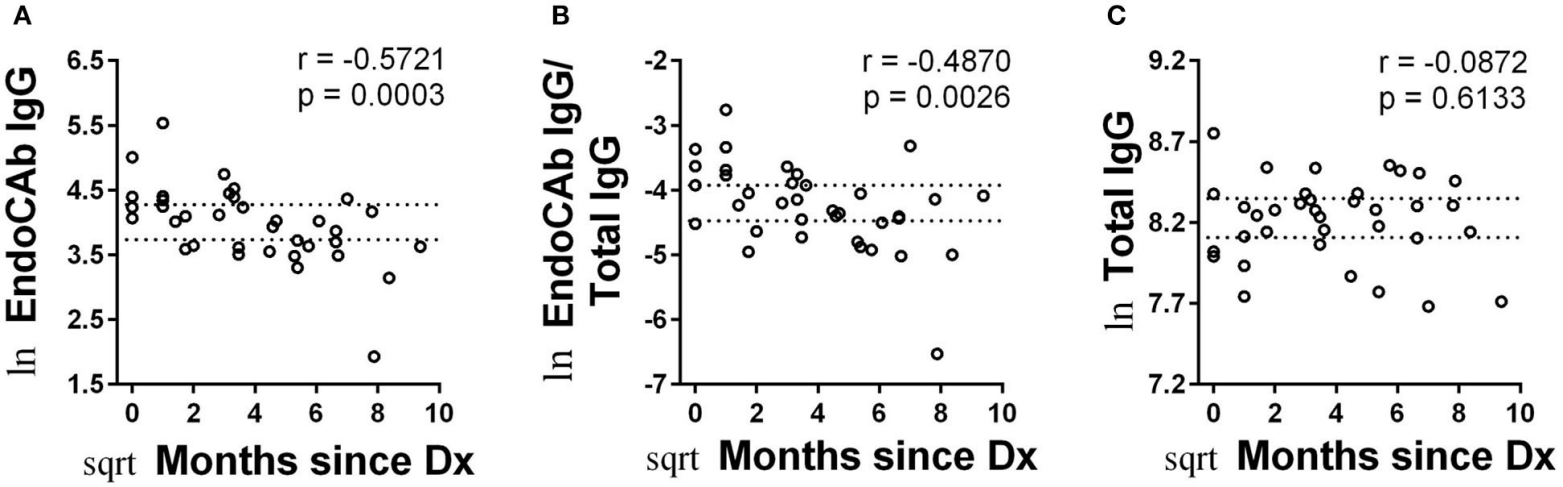
Abundance of EndoCAb IgG decreased with time since diagnosis. **(A,B)** EndoCAb IgG levels as well as the ratio of EndoCAb IgG to total IgG decrease with months since diagnosis for T1D subjects. **(C)** Total IgG levels were not significantly associated with months since diagnosis. Dotted lines represent upper and lower 95% confidence intervals based on control subjects for each analyte and are included for reference. Data were normalized using natural logarithm (ln) and square root (sqrt) transformations.

Endotoxin core-specific antibodies (EndoCAbs) provide a sensitive measurement of B cell-driven responses to LPS with elevations and decreases indicating dynamic exposure to endotoxin [reviewed in (19)]. Varying EndoCAb levels have also been associated with autoimmunity (20, 21). We found no changes in abundance of EndoCAbs IgA, IgG, and IgM comparing T1D and control (Figures 2A–C). At the same time, we examined total IgG, IgA, and IgM, whose levels reflect global immune experience and activation. We observed no difference between controls and T1D for total IgG and total IgA levels (Figures 2D,E). However, we found total IgM was significantly, though modestly, reduced compared to controls. (Figure 2F). We also observed no difference between controls and T1D for proportion of EndoCAbs IgG, -A, and -M to total immunoglobulins G, A, and M (Figures 2G–I). Interestingly, EndoCAb IgG levels as well as the ratio of EndoCAb IgG to total IgG decreased with months since diagnosis in T1D (Figures 3A,B). This was not the case for total IgG levels (Figure 3C), nor for total IgM, IgA, and EndoCAbs IgA and IgM (data not shown). In total, aside from a modest elevation of EndoCAb IgG around diagnosis, there is little evidence of systemic atypical endotoxin exposure in T1D. Furthermore, previous immunological exposure gauged by circulating IgG and IgA levels appears comparable between the two groups, while reduced IgM in T1D may suggest somewhat dampened B cell activation.

### Circulating Levels of sCD14 Are Significantly Elevated in T1D, While Elevated CRP Levels Are Specific to T1D Females

**Figure 4:**
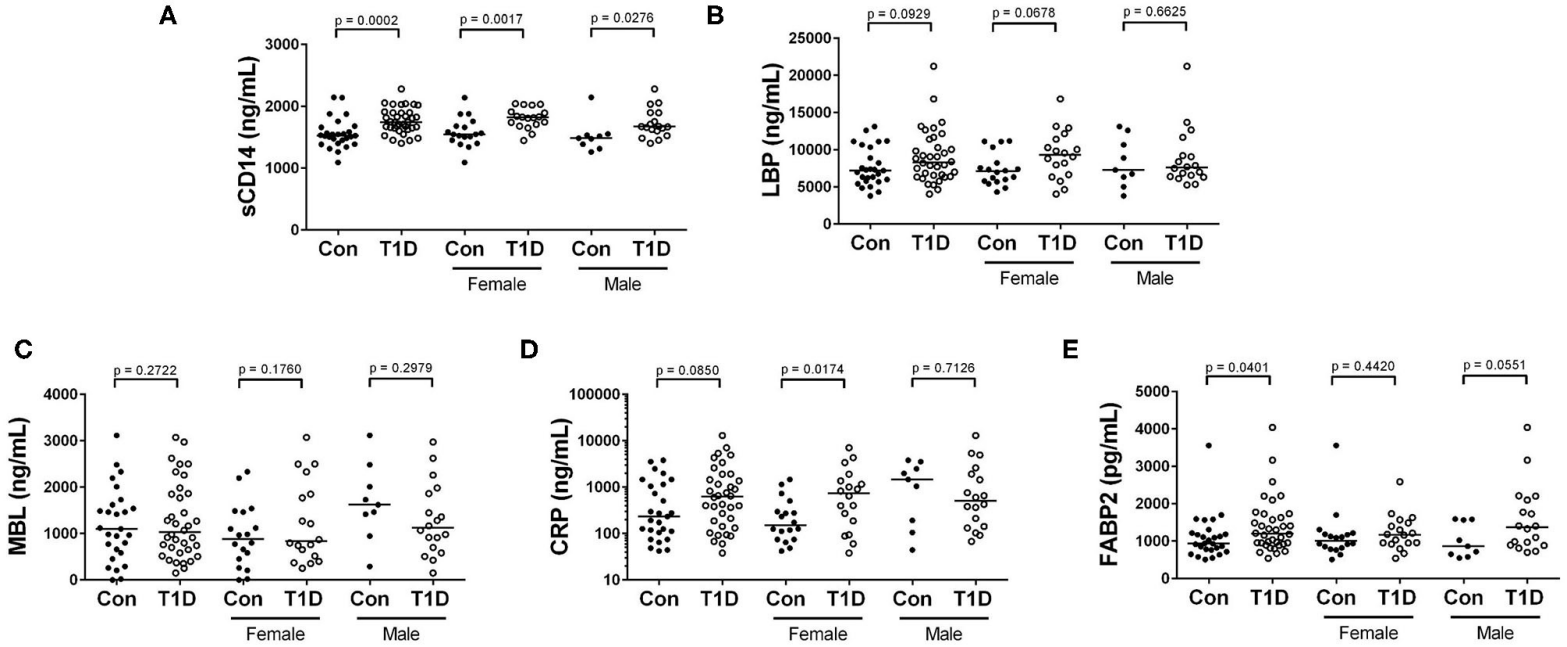
Elevated sCD14 and FABP2 in T1D subjects, and heightened CRP among female T1D subjects. **(A)** We observed significantly heightened sCD14 levels among T1D subjects compared to control. **(B,C)** Lipopolysaccharide binding protein (LBP) and mannose binding lectin (MBL) were relatively similar between the two groups and revealed no significant differences. **(D)** C reactive protein (CRP) levels were relatively similar when comparing total T1D subjects, yet when stratifying by sex, female T1D subjects were significantly elevated in relation to female controls. **(E)** Fatty acid binding protein 2 (FABP2) levels were significantly elevated in T1D subjects. Bars represent median values.

Acute-phase proteins such as lipopolysaccharide binding protein (LBP), mannose binding lectin (MBL) c-reactive protein (CRP), and soluble CD14 (sCD14) rapidly change in abundance during inflammatory responses (22, 23). Fatty acid binding protein 2 (FABP2) is a marker of intestinal epithelial damage and integrity and can be used as a proxy measure for the health of the intestinal epithelium (24). We observed significantly increased sCD14 among T1D subjects, while levels of LBP and MBL were unchanged between the two groups (Figures 4A–C). CRP levels were also similar for total T1D and controls, yet upon stratifying into male and female, we found elevated CRP levels in female T1D subjects compared to control, while males T1D subjects and controls were similar (Figure 4D). Finally, we observed slightly elevated FABP2 levels in T1D subjects (Figure 4E).

### 25 (OH) Vitamin D and Vitamin D Binding Protein Levels Are Similar Among Control and T1D Subjects

**Figure 5:**
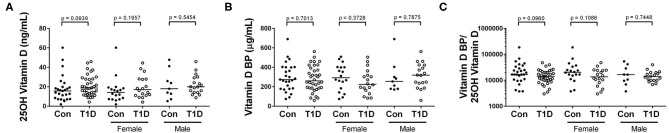
25(OH) vitamin D and vitamin D BP levels are similar between control and T1D subjects. **(A,B)** We observed no difference in abundance of 25-hydroxy vitamin D (25(OH) vitamin D) nor vitamin D binding protein (BP) among T1D subjects and control. **(C)** Additonally, the ratio of vitamin D BP to 25(OH) vitamin D was similar among the two groups **(C)**. Bars represent median values.

Vitamin D is a powerful immunomodulatory agent involved in assorted immune pathways and implicated in several diseases including autoimmunity (25). While multiple factors govern its abundance and availability, one method by which its circuiting levels are managed is through the vitamin D binding protein (BP) (26). Due to the importance of this pathway in immunity, we sought to investigate vitamin D and vitamin D BP levels in T1D. We observed no difference in 25(OH) vitamin D and no difference in vitamin D BP levels among control and T1D subjects (Figures 5A,B). Furthermore, we observed no difference in ratio of vitamin D BP to 25(OH) vitamin D among control and T1D subjects (Figure 5C). This ratio indicates systemic accessibility to vitamin D. In combination, we found no signs that vitamin D levels differ between controls and T1D.

### Leptin, Adiponectins, and HGH Levels Are Similar Among Control and T1D Subjects

**Figure 6:**
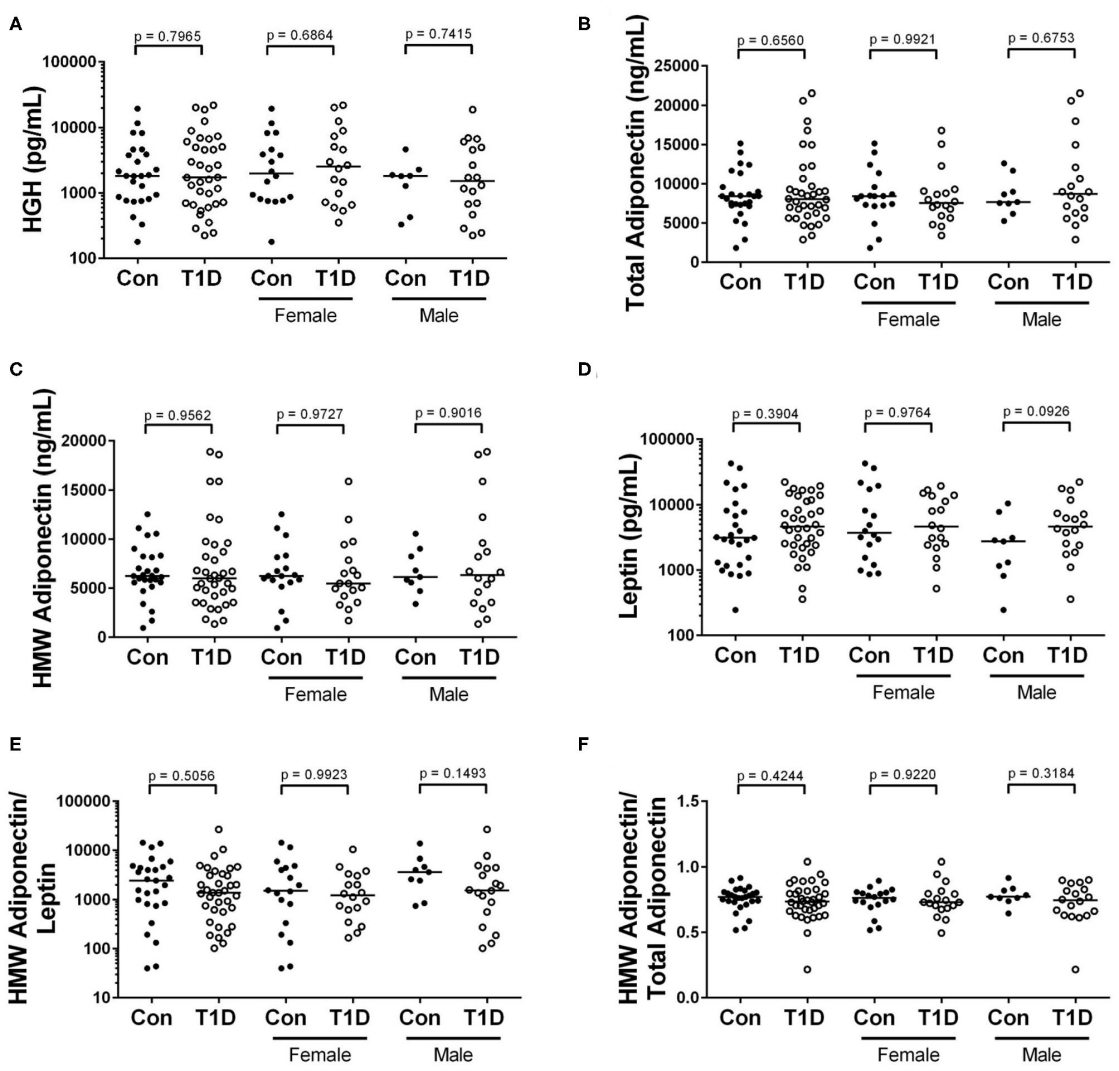
No difference in levels of adipokines and HGH when comparing control and T1D subjects. **(A)** We found no difference in abundance of human growth hormone (HGH) among T1D subjects and controls. **(B–D)** Similarly, the levels of total adiponectin, high molecular weight (HMW) adiponectin, and leptin were relatively equivalent when comparing the two groups. **(E,F)** Finally, the ratios of HMW adiponectin to leptin and HMW adiponectin to total adiponectin were roughly equivalent among the groups. Bars represent median.

Energy homeostasis involves cross-talk between multiple tissues and organ systems. For example, adipokines like leptin and adiponectin are derived from adipocytes and involved in insulin sensitivity and immune modulation (27, 28). Additionally, human growth hormone (HGH) is secreted from the pituitary gland and drives growth and energy demand in all tissues (29). We observed no significant difference in levels of circulating HGH, total adiponectin, high molecular weight (HMW) adiponectin, and leptin among control and T1D subjects (Figures 6A–D). Furthermore, as the ratio of HMW adiponectin to total adiponectin and to leptin reveal propensities to insulin resistance and inflammation (30, 31), we also examined them. These ratios were unchanged among T1D subjects and controls (Figures 6E,F).

### Elevated Levels of IL-18BP Isoform a Found in T1D, While Levels of IFN-γ, TNF-α, IL-10, IL-7, IL-18, IL-17A, and MDC Are Similar to Controls

**Figure 7:**
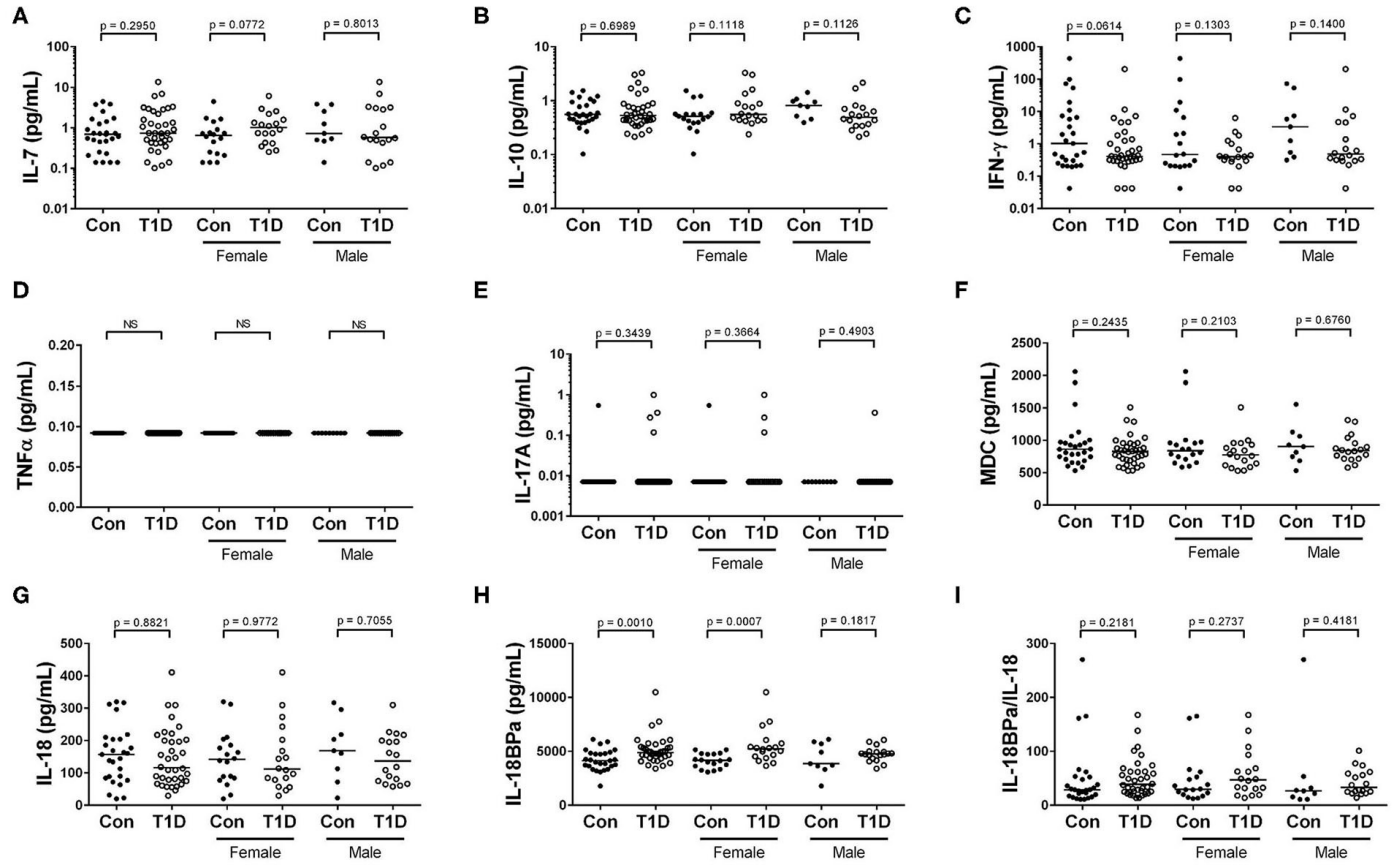
Elevated IL-18BPa among T1D subjects, while other cytokines show similar abundance. **(A–C)** We observed that IL-7, IL-10, and IFN-γ levels were more-or-less similar among T1D subjects and controls. **(D,E)**. We were unable to detect TNF α and IL-17A in the majority of our subjects. **(F)** The chemokine macrophage derived chemokine (MDC) was similarly abundant among T1D subjects and controls. **(G,H)** While IL-18 levels were roughly equivalent between the two groups, elevated IL-18BPa levels were observed among the T1D group in relation to control. **(I)** Although IL-18BPa was elevated, the ratio of IL-18BPa to IL-18 was similar among T1D subjects and controls. Bars represent median.

Cytokine and chemokines control cellular activation, maturation, migration and survival, and elevated levels of these can indicate active infections and inflammatory processes. One difficulty in the analysis of many circulating cytokines is their relative scarcity in plasma. We sought to overcome this by utilizing high-sensitivity assays which are capable of detecting analytes to the low femtogram per mL level. We analyzed plasma for circulating levels of IFN-γ, TNF-α, IL-10, IL-17A, and IL-7 using high sensitivity assays. We observed no differences in levels of circulating IL-7, IL-10, and IFN-γ among controls and T1D (Figures 7A–C). For TNF-α, we were unable to detect any quantity in circulation from either our controls or T1D cohort (Figure 7D). Similarly, IL-17A was mostly undetectable in the plasma, with only 1 control and 4 T1D subjects having detectable levels (Figure 7E). Among the more abundant circulating targets are macrophage derived chemokine (MDC) as well as IL-18 and its inhibitor, IL-18BPa. These could be detected using standard ELISA approaches. Neither MDC nor IL-18 levels were different among T1D and control subjects (Figures 7F,G). However, IL-18BPa levels were elevated in T1D compared to controls yet the ratio of IL-18BPa to IL-18 among T1D and controls was similar (Figures 7H,I). In total, we found no sign of elevated cytokine in T1D subjects, thereby providing no evidence of disease-specific immune responses in progress.

### Higher C—Peptide Levels Early After Diagnosis Are Associated With Lower Levels of Adiponectin

**Figure 8:**
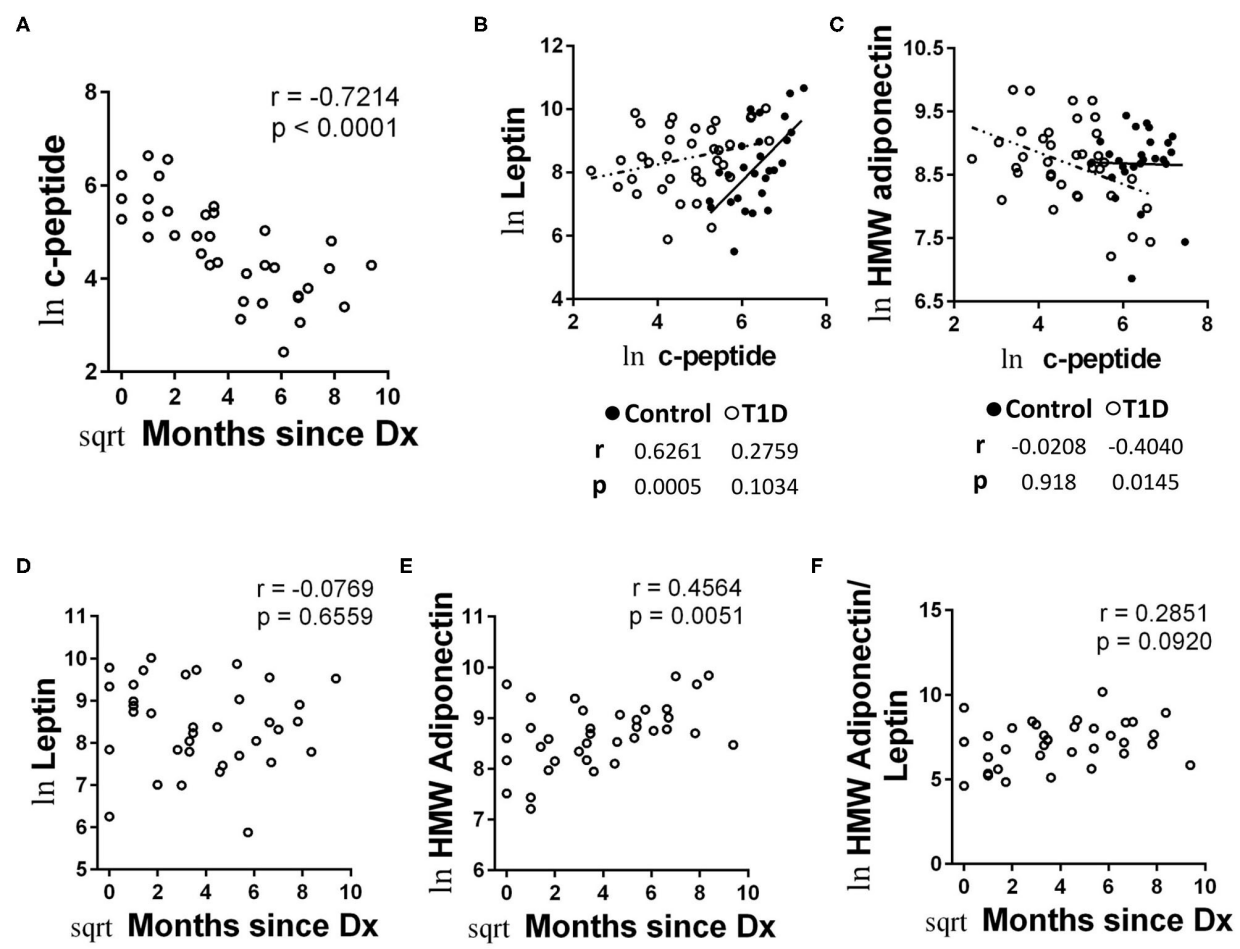
Unique associations with c-peptide and adipokines among T1D subjects and increasing HMW adiponectin with time since diagnosis. **(A)** C-peptide levels rapidly decrease with months since diagnosis. **(B)** Leptin levels were positively and significantly associated with c-peptide levels in controls, while the relationship was weaker among T1D subjects and did not reach significance. **(C)** HMW adiponectin was not significantly associated with c-peptide levels among controls, while T1D subjects possessed a negative and significant association between HMW adiponectin and c-peptide. **(D)** Leptin levels were not significantly associated with time since diagnosis amount T1D subjects. **(E)** HMW adiponectin levels increase with time since diagnosis among T1D subjects. The results were similar for total adiponectin (data not shown). **(F)** The ratio of HMW adiponectin to leptin was weakly associated with time since diagnosis, but did not reach significance. Data were normalized using natural logarithm (ln) and square root (sqrt) transformations.

C-peptide levels offer a direct connection to beta cell function. Although extremely low in the majority of cases, all of our T1D subjects had measurable c-peptide. To explore c-peptide dynamic/beta cell function more fully, first we compared c-peptide levels with time since diagnosis. Using untransformed data, we found what appeared to be a rapid decrease in c-peptide levels with months since diagnosis appearing as an exponential decay (Supplemental Data 3). Transforming the data for parametric statistical analysis revealed a significant decrease in c-peptide levels with time since diagnosis (Figure 8A). This pattern has been demonstrated previously (32).

We then compared the values from metabolic factors and autoantibodies with c-peptide levels for correlative strength. We observed no significant correlations between autoantibody and c-peptide levels (data not shown). We did find significant correlations with c-peptide levels among the adipokines, however. Namely, c-peptide was strongly and significantly associated with leptin levels among controls, yet the association was relatively weak among T1D subjects and did not reach significance (Figure 8B). Alternatively, levels of HMW adiponectin were negatively associated with c peptide levels among T1D subjects, reaching a significance of *p* = 0.0145 (Figure 8C). This was in opposition to the control group, in which c-peptide and HMW adiponectin levels appeared to possess no association (*r* = −0.0208, *p* = 0.918; Figure 8C). Lastly, we were curious how adipokine levels may change with time since diagnosis, due to their association with c-peptide levels. While we noticed no significant association with leptin levels and months since diagnosis (Figure 8D), we did observe the both total adiponectin (data not shown) and HMW adiponectin appeared to increase with months since diagnosis (Figure 8E). Finally, the association between months since diagnosis and the ratio of HMW adiponectin to leptin was relatively weak and not significant (Figure 8F).

## Discussion

### Cytokines

One general takeaway from our results is an overall lack of demonstrably unique cytokine responses among diabetics compared to controls. Among previous studies, our findings are in agreement with some reports, but not others. For example, TNF-α has been found to be elevated (33–35), unchanged (36–39), as well as decreased (40) in type 1 diabetes. Here, using a high-sensitivity assay with a limit of detection of 130 fg/mL, we were unable to detect circulating TNF-α in any of our subjects. Similarly, IL-17A was mostly undetectable in plasma. Indeed, using an assay with a sensitivity of 10 fg/mL only 1 control and 4 T1D subjects possessed measurable IL-17A levels. As with TNF-α, IL-17A has been found to be both elevated (40–42) and unchanged among T1D subjects (36, 43).

Unlike TNF-α and IL-17A, we were able to observe measureable MDC, IL-7, IL-10, IL-18, and IFN-γ in the majority, if not all, of our subjects. Nevertheless, when comparing controls and T1D, the levels of these cytokines and chemokine were relatively similar. Unchanged IL-7 levels have been reported by Alnek and associates (36), while unchanged IL-10 and IFN-γ levels have been reported by others (39, 44). Contrasting these findings are reports of reduced (40) or elevated (33, 36, 45) IL-10 among T1D subjects, as well as elevated IFN-γ (33). While we observed no difference in IL-18 levels between our cohorts, we have previously observed a modest IL-18 elevation in T1D associated with hyperglycemia (46). Elevations in IL-18 in T1D have also been reported by others (47–49). In sum, circulating cytokine abundance in type 1 diabetes remains unresolved being awash in conflicting reports. Such widely variable results could represent a bevy of type I and/or type II errors due to sampling bias, flaws in experimental design, and/or technical differences. For example, among the studies cited for the analysis of TNF-α and including ours, no group used the same methodology. For subject characteristics, six of the cited TNF-α studies focused on juveniles, while two investigated a broader age range. These and other aspects of experimental design may explain these disparate conclusions. Additional variables are that glycemic excursions and ketoacidosis can impact abundance of several circulating cytokines (50–52). Lastly, the episodes that could create detectable cytokine differences may only occur well-before diagnosis, during discrete and brief windows of time.

Although not a cytokine *per se*, we observed elevated IL-18BPa among T1D subjects and this elevation appeared most prominent in females. IL-18BP is a natural inhibitor of IL-18 function (53). We previously examined IL-18BP using a commercially available assay which did not differentiate isoforms (46). Using that previous approach, we observed similar concentrations between diabetics and controls. Interestingly, although IL-18BPa was elevated in our current cohort, the proportion of IL-18BPa to IL-18 was similar among controls and diabetics. This is consistent with other reports which demonstrate IL-18BP and IL-18 increasing and decreasing concordantly (54, 55), except in unique pathologies (56). While a provocative finding, the elevation of IL-18BPa raises the questions of source and cause. To that end, we found no correlative association with IFN-γ levels (data not shown), which could be expected if a feedback loop was observable in circulation (57).

### Vitamin D

Reduced vitamin D levels have been hypothesized as a risk factor for the development of type 1 diabetes and other forms of autoimmunity [reviewed in (58)]. These reductions are posited to have a negative effect on both innate and adaptive immunity (ibid.). Here, we report no significant differences among T1D subjects and controls for vitamin D BP and 25(OH) vitamin D levels, as well as for the ratio of vitamin D BP to 25 (OH) vitamin D, an estimate vitamin D accessibility. Our results stand in contrast to previous reports of reduced vitamin D binding protein in type 1 diabetes (59), and contribute to the unsettled issue of 25(OH) vitamin D levels among type 1 diabetics as either reduced (60, 61) or unchanged (62). Finally, many of our T1D subjects and controls could be described as deficient for vitamin D, in line with previously published results (63). We are wary of assigning this designation for two reasons. First, seasonal variation can effect vitamin D levels and our subjects were sampled through Fall—early Spring sequence of time, when solar exposure would be limited. Second, there is some indication that designations of “deficiency” may be over-applied and should be used more judiciously [see (64)].

### Pathogens

Our query of previous exposure to a limited range of pathogens showed both similarities and differences for relatively common viruses. Firstly, adenovirus and parainfluenza 1/2/3 responses were generally similar between T1D and control. Both adenovirus and the parainfluenza group are common childhood pathogens and would be expected to be highly seroprevalent. None are known to form chronic infections, and, to our knowledge, have not been associated with T1D incidence.

Alternatively, Coxsackievirus has a long history of association with T1D [reviewed in (65)]. We found Coxsackievirus IgG prevalence was also similar between groups, demonstrating equivalent exposure and responses among T1D and controls. Therefore, we have no evidence to implicate Coxsackievirus in T1D pathogenesis. One unexpected finding was the significantly higher prevalence of Coxsackievirus/echovirus IgG in females rather than males. Sex-based differences in viral responses are well-documented (66), and this is likely one such example.

The Coxsackievirus assay we used contains a mixture of VP1 proteins from Coxsackievirus B1, B3, and B5 (personal communication from manufacturer). The responses measured would presumably be limited to those strains. However, we ran echovirus IgG assays at the same time for comparison and observed nearly perfect correlation between the two assays. This echovirus assay utilizes VP1 proteins from echovirus E6 and E9 (personal communication from manufacturer). Our concordant results between the Coxsackievirus and echovirus assays denotes broad cross-reactivity among enteroviral VP1. Such VP1 cross-reactivity has been reported previously (67, 68). From these results, it's likely that our Coxsackievirus/echovirus IgG positive subjects bind VP1 proteins from other members of *Enterovirus B*, if not other species in the genus.

In contrast to the other pathogens, herpesviruses are all capable of forming chronic infections and, thus, have profound impact on the immune system. Prevalence of EBV and CMV were both lower among T1D compared to controls. This reached a modest level of significance for EBV and was most prominent among females. Unexpectedly, HSV1 prevalence was higher in T1D and those positive were exclusively male. The meaning behind these changes in herpesvirus prevalence is not known, although they are certainly provocative due to the involvement of both CMV and EBV in autoimmunity (69, 70). Due to the tightly-matched age and localized residence of our group, it would expected that viral exposure and response should be similar. Whether these reductions reflect compromised immune responses, unequal exposures, or a random outcome of sampling is unknown. To resolve this ambiguity, we are planning to test additional, larger groups for these and other viruses.

### Systemic Endotoxin and Acute Phase Responses

Microbially-based etiologies with associated intestinal permeability have become relatively popular causal hypotheses for autoimmunity and type 1 diabetes in general (71). From our analysis, we found limited supportive data indicating elevated systemic endotoxin or other microbial factors among T1D subjects. Importantly, we observed no significant differences in relative abundance of EndoCAb antibodies using a commercial assay which measures endotoxin-targeting immunoglobulins targeting four groups of bacteria (e.g., *Escherichia coli, Klebsiella, Pseudomonas* and *Salmonella*—manufacturer's communication). In contrast to our findings, at least one previous report has shown substantial reductions in EndoCAb IgG levels among T1D subjects compared to controls (34). Similarly to the EndoCAbs, we found MBL and LBP levels were not significantly different between our groups. While more will be said of MBL below, our findings with LBP are in line with a previous report showing similar LBP levels among T1D and control (72), while in opposition to a report showing reduced LBP among T1D subjects (34). Lastly, we observed no significant difference between control and T1D subjects for CRP, a protein with anti-microbial functions denoting generalized inflammation (73). However, upon stratifying by sex, female T1D subjects appeared elevated for CRP while male T1D subjects were relatively similar to control. As already noted, female T1D subjects were also more prominently elevated for IL-18BPa. Yet, the connection between CRP and IL-18BPa is unclear in the absence of a causal intermediary. At this point, we have no evidence to advance or dismiss a microbial origin for these sex-specific responses.

Regarding MBL, previous reports have shown it to be increased in T1D, specifically when comparing high-producing MBL genotypes (74), or when comparing T1D subjects with at-risk siblings (75). As our study did not involve genetic recognition of MBL genotypes, nor at-risk siblings, we were unable to explore these dimensions. We therefore cannot rule out similar genetically-based differences.

Circulating FABP2 levels can be used as sign of intestinal epithelial damage (76, 77), with elevation being suggestive of increased systemic exposure to gastrointestinal denizens and their byproducts. Although we observed a significant elevation in FABP2 among T1D, this was a very modest increase, and, upon stratification by sex, was mostly associated with males. This could be interpreted as a sign of minor intestinal epithelial disturbance among juvenile male type 1 diabetics. However, a much larger cohort should be examined to pursue this novel finding robustly.

In contrast to these negative or equivocal results, two of our findings could support the concept that elevated microbial exposure is associated with T1D. One, we observed significantly elevated sCD14 levels among T1Ds, contrasting a previously report of unchanged sCD14 in T1D (34). While sCD14 functions to direct LPS responses in circulation (78–80), its release is also a sign of general monocyte activation (81). Thus, elevations are difficult to causally define. Second, we found elevated levels of EndoCAb IgG early following diagnosis. This suggests a heightened humoral response to endotoxin in the events leading to and immediately following clinical presentation of disease. As one can only extend a cross-sectional analysis so far, longitudinal analysis of at-risk subjects is the logical follow-up from these findings.

### Adipokines

Our examination of the adipokines and HGH revealed a relative equivalence in abundance among control group and T1D. This is contrary to previous reports showing elevated leptin and adiponectin (44, 82), as well as HGH (83, 84), in T1D. Although relative abundance was equivalent, we did observe unique associations between the adipokines and c-peptide levels among the T1D group. Both leptin and adiponectin are thought to influence insulin sensitivity (85, 86). Regarding leptin, we observed a strong positive correlation between c-peptide levels and leptin levels in control group, yet the association was relatively weak in T1D. Furthermore, we observed little change with leptin levels or its ratio with HMW adiponectin over time since diagnosis. Thus, in an environment of highly reduced natural insulin secretion, the fine-tuned relationship between leptin and insulin may be dysregulated. Alternatively, HMW adiponectin was negatively correlated with c-peptide among the T1D group, while control group showed no definite relationship between the two. Thus, the T1D subjects with the highest c-peptide had the lowest HMW adiponectin levels. Since the subjects with high c-peptide were relatively early from diagnosis, there could be a type of adiponectin-associated insulin resistance present at diagnosis among some subjects. The idea of insulin resistance early following diagnosis has been suggested previously (87), yet a formal mechanism is wanting. Nevertheless, this does not appear to be a lasting condition as we observed that adiponectin abundance increased with time since diagnosis.

## Conclusion

We investigated several prevailing hypotheses into the pathogenesis of T1D by screening human plasma for the abundance of multiple pertinent targets. Our study was not without limitations, however. First, it was confined to measurements in plasma due to the inaccessibility of the target tissues. The immune response driving beta cell destruction is likely subtle, judging from what is known of human insulitis (88). Thus, it may be that peripheral measurements are incapable of detecting localized tissue-specific responses. Second, as has been mentioned, the study was cross-sectional. Longitudinal analysis of subjects may provide a more telling examination of such dynamic factors. Third, it could be that the majority of these responses occur during the prodromal period, and that following diagnosis, the major pathogenic events are no longer as easily detectable. Fourth, our T1D group possessed higher mean BMI than our control group. Our results may be confounded by this difference. Fifth, being focused on environmental factors, our analysis did not include an evaluation of HLA class I and II. Sixth, while we attempted to survey the literature to pick appropriate targets to test these hypotheses, it may be that we selected inappropriately and that other soluble factors may properly reveal such relationships. To that end, we encourage continued investigation.

With these limitations in mind, we found that among tightly age-matched juvenile T1D subjects and heathy controls, there are few measurable differences in abundance of disease-associated circulating soluble factors. While we observed enticing hints of potential causality, we found scant strong evidence supporting any of the causal hypotheses explored. Our inability to observe strong evidence could be due to its non-existence or could be due to the limitations described above. To fully unravel the contribution of the environment to the disease, additional study of subjects at-risk of developing T1D and soon after diagnosis is required.

Finally, of the significant differences we observed, sCD14 and IL-18BPa are most statistically robust. Being produced chiefly by monocytes and macrophage, these elevations suggest heightened activation of monocytes and monocyte-derived cells. It remains to be determined if these elevations are associated with T1D pathogenesis and what specific stimuli are driving these changes.

## Data Availability Statement

All datasets presented in this study are included in the article/Supplementary Material.

## Ethics Statement

The studies involving human participants were reviewed and approved by Institutional Review Board of the University of Nebraska Medical Center. Written informed consent to participate in this study was provided by the participants' legal guardian/next of kin.

## Author Contributions

RH and KO performed the experiments and analyzed the data, RH processed samples, KO obtained consent and acquired samples, MC, EE, and MF patient selection and sample acquisition, RH wrote manuscript, and RH and NS designed the experiments. All authors contributed to the article and approved the submitted version.

## Conflict of Interest

The authors declare that the research was conducted in the absence of any commercial or financial relationships that could be construed as a potential conflict of interest.
