# Supplementary figures and images for: Confirmation and Identification of Biomarkers Implicating Environmental Triggers in the Pathogenesis of Type 1 Diabetes
Source: Front Immunol. 2020 Sep 15;11:1922. doi: 10.3389/fimmu.2020.01922 (PMC7523316; doi:10.3389/fimmu.2020.01922)

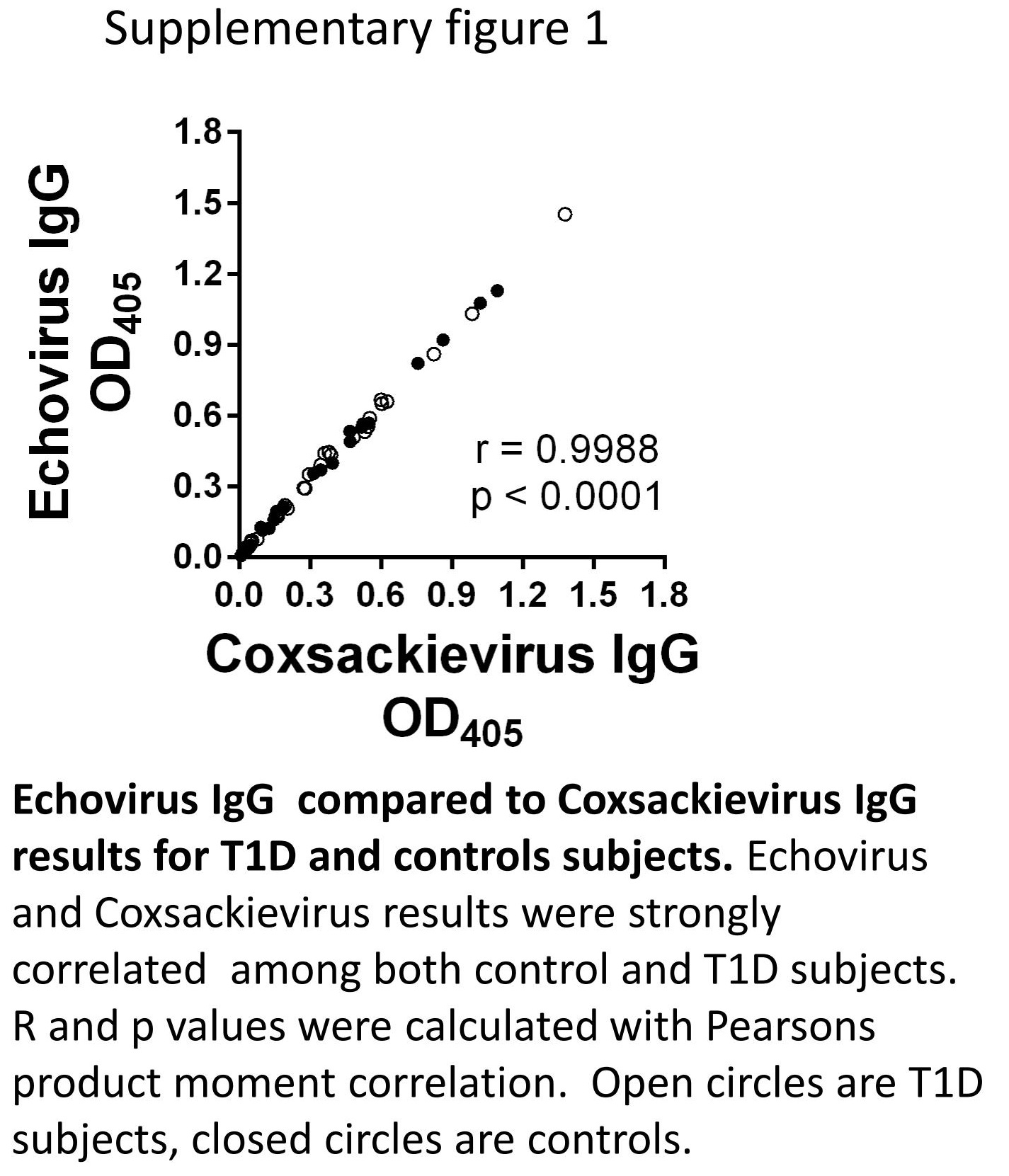

Supplement: Supplementary file 2 [file Image_1.jpg]

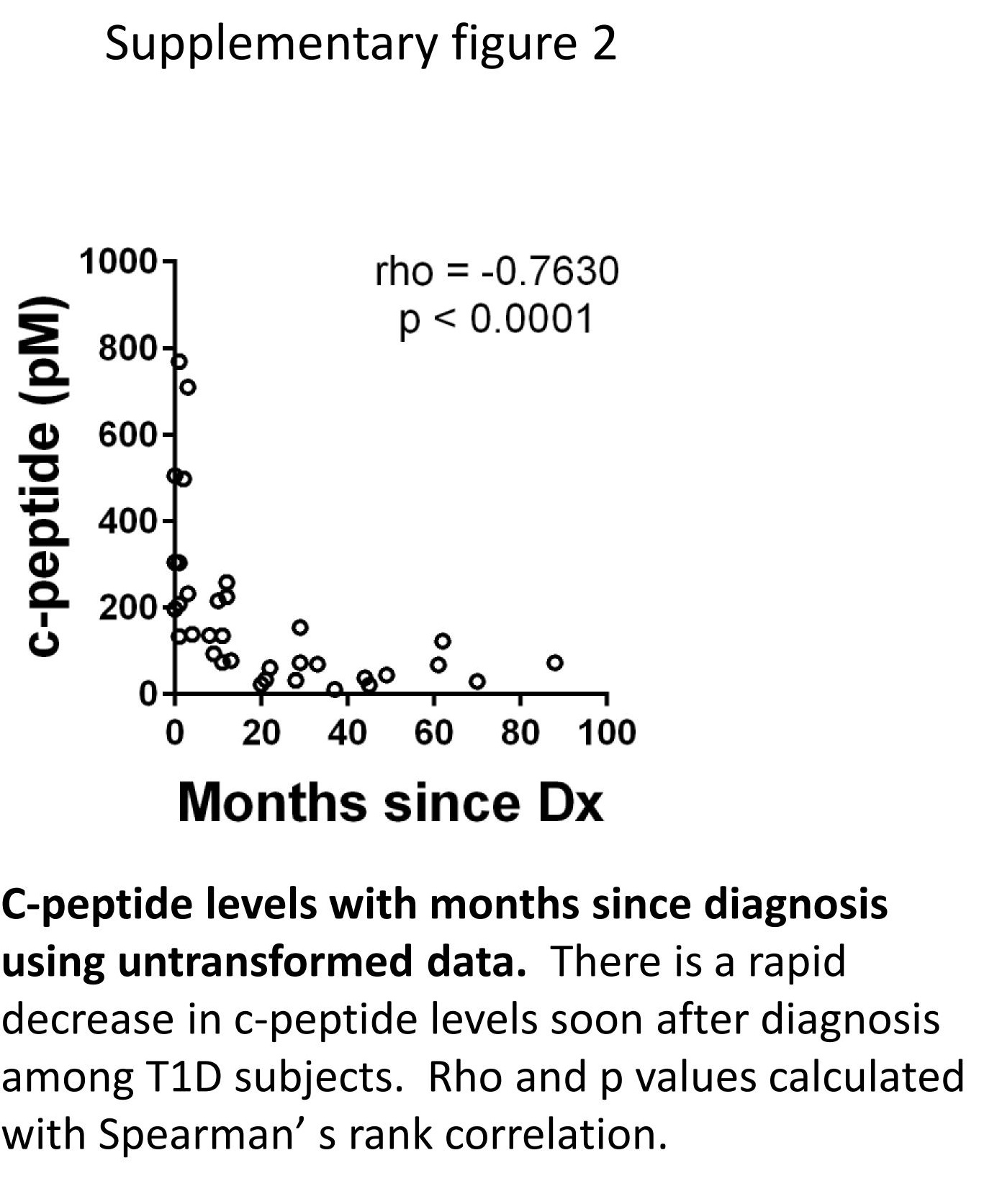

Supplement: Supplementary file 3 [file Image_2.JPEG]
